# Network Models and Simulation Analytics for Multi-scale Dynamics of Biological Invasions

**DOI:** 10.3389/fdata.2022.796897

**Published:** 2022-02-07

**Authors:** Abhijin Adiga, Nicholas Palmer, Young Yun Baek, Henning Mortveit, S. S. Ravi

**Affiliations:** ^1^Biocomplexity Institute and Initiative, University of Virginia, Charlottesville, VA, United States; ^2^Department of Engineering Systems and Environment, University of Virginia, Charlottesville, VA, United States; ^3^Department of Computer Science, University at Albany—SUNY, Albany, NY, United States

**Keywords:** invasive species, network models, diffusion processes, multi-scale networks, simulation analytics, spatial networks

## Abstract

Globalization and climate change facilitate the spread and establishment of invasive species throughout the world via multiple pathways. These spread mechanisms can be effectively represented as diffusion processes on multi-scale, spatial networks. Such network-based modeling and simulation approaches are being increasingly applied in this domain. However, these works tend to be largely domain-specific, lacking any graph theoretic formalisms, and do not take advantage of more recent developments in network science. This work is aimed toward filling some of these gaps. We develop a generic multi-scale spatial network framework that is applicable to a wide range of models developed in the literature on biological invasions. A key question we address is the following: how do individual pathways and their combinations influence the rate and pattern of spread? The analytical complexity arises more from the multi-scale nature and complex functional components of the networks rather than from the sizes of the networks. We present theoretical bounds on the spectral radius and the diameter of multi-scale networks. These two structural graph parameters have established connections to diffusion processes. Specifically, we study how network properties, such as spectral radius and diameter are influenced by model parameters. Further, we analyze a multi-pathway diffusion model from the literature by conducting simulations on synthetic and real-world networks and then use regression tree analysis to identify the important network and diffusion model parameters that influence the dynamics.

## 1. Introduction

### 1.1. Background and Motivation

Many natural- and engineered complex systems can be represented as systems of interconnected networks (Buldyrev et al., 2010; Barrett et al., 2013; Bashan et al., 2013). Such representations are very effective at capturing relationships of different types and at different scales between the entities comprising the complex system. There are several classes of such networks depending on the application, including multi-layer networks, multiplex networks, and network of networks, to name just a few. We refer the reader to Kivelä et al. (2014) for a comprehensive list. Here, we study modeling of complex networks arising in the context of multi-scale spatial dynamical processes, such as biological invasions, spread of infectious diseases, and built infrastructure (Balcan et al., 2009; Serrano et al., 2009; Walpole et al., 2013), our focus mainly being on the properties of dynamical processes over such networks.

In recent years, there has been a big thrust toward developing cyberinfrastructures to address problems related to resilient and sustainable agriculture (USDA-NSF, 2017, 2021; Microsoft, 2020). One of the greatest threats to biodiversity and food security is the increasing rate of biological invasions (Pimentel et al., 2005; Crowl et al., 2008; Pyšek and Richardson, 2010). The annual economic costs arising from environmental damages and the losses caused by invasive species run into billions of dollars (Diagne et al., 2021). Understanding the role of natural processes and human activities in the spatio-temporal spread of biological invasions is of utmost importance (Cunniffe et al., 2015).

**Figure 1:**
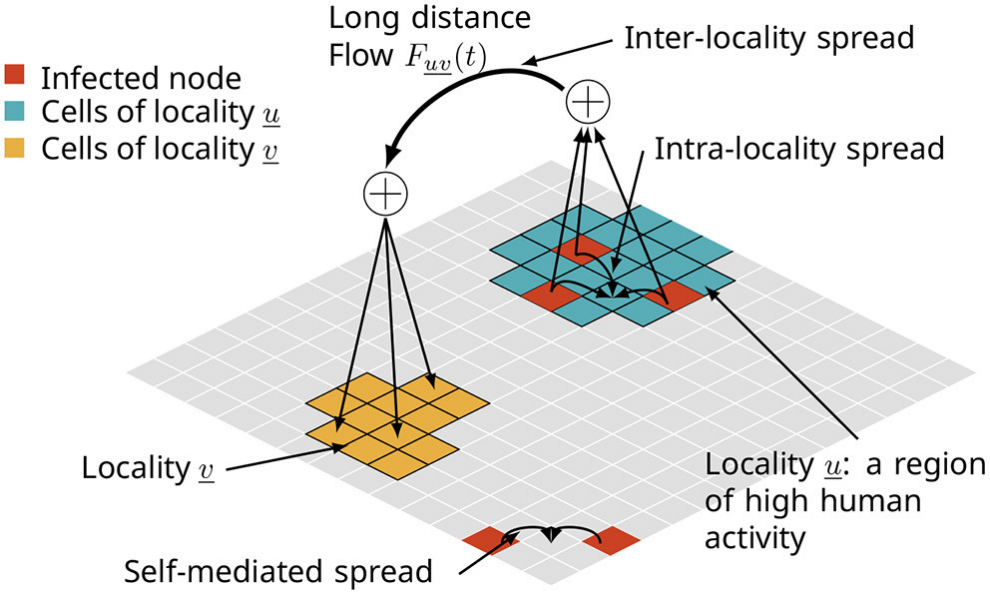
A multi-pathway network model of a spatial diffusion process in the context of biological invasions. The spread takes place at multiple spatial scales, such as (i) one cell to adjacent cells due to self-mediated spread, (ii) within a locality of high human activity, and (iii) long distance jumps facilitated by trade and travel.

A variety of models for biological invasions have been proposed with the goal of studying these complex phenomena at appropriate levels of resolution and fidelity (Pitt et al., 2009; Carrasco et al., 2010; Smolik et al., 2010; Fitzpatrick et al., 2012; Robinet et al., 2012; Ferrari et al., 2014; Chapman et al., 2015; Douma et al., 2016; McNitt et al., 2019; Venkatramanan et al., 2020). In many of these works, biological invasions have been viewed as propagation processes over multi-scale networks. As depicted in Figure 1, there are various pathways or modes of dispersal. The spreading ability of the invasive organism, environmental factors (e.g., winds, ocean currents, and suitable environment), and anthropogenic factors (e.g., production and trade of host crops, and tourism) are among the primary pathways. These pathways affect the spread at multiple spatial and temporal scales. For example, the rate and pattern of self-mediated spread can be significantly different from that of human-assisted spread (Davis et al., 2001; Hoffmann and Courchamp, 2016). The functions or mechanisms that govern the process can vary within and across scales; the flying ability of a pest determines how far it can naturally spread, while trade of host crops and plant material can facilitate long distance spread. Accordingly, there are potentially several ways to control the phenomenon ranging from application of pesticides (farm-level) to trade restrictions (administrative-level).

While the models referenced above have been designed and analyzed from the perspective of biological invasions, there has been very little work on exploring the connection to well-known formal frameworks developed in network science. A typical modeling effort uses networks to represent movements of species based on data or assumptions regarding the processes involved (or both). The resulting networks are analyzed using basic structural properties such as degree distribution and betweens centrality. Further, suitable diffusion models are used to simulate the spread over this network. It is important to understand these models from a network science perspective in order to (i) make robust predictions, (ii) rigorously address key aspects such as calibration, validation, verification, sensitivity analysis, and uncertainty quantification, and (iii) leverage state-of-the-art algorithms to address important problems pertaining to control, monitoring, and various inference problems in the field of network dynamical systems. The aim of this work is to provide the first steps in developing the foundations for these models. Through such formal grounding and abstractions one may also be able to take advantage of existing mathematical and computational theory, including computational paradigms facilitating efficient mapping of models onto high-performance software implementations that exploit modern hardware.

In this paper, we develop an abstract model for multi-pathway spatial processes. For this model, we characterize complex spatial diffusion processes using structural properties of the underlying network. Next, we provide an analysis of the roles of the different pathways on the rate and pattern of spread by applying a complex multi-pathway diffusion model proposed in McNitt et al. (2019), which we refer to as SPREAD (which stands for Simulation tools for Pest Risk analysis accounting for Ecological and Anthropogenic Drivers). Finally, we provide analyses of structural and dynamical properties of networks through the lens of machine learning algorithms to identify the primary drivers of spread. A more detailed description of our contributions is as follows.

### 1.2. Contributions

#### 1.2.1. A Model for the Multi-Pathway Spatial Network

We define a grid-based model called MPSN for synthetic spatial networks which captures the multi-scale nature of the spread process. The synthetic network is composed of nodes of a grid and metanodes called localities, each of which consists of a unique set of contiguous grid nodes. Its edge set is the union of the edge sets of three different graphs, each corresponding to a different pathway of spread as in Figure 1. The main purpose of proposing such a model is to bridge the gap between simple static network models like Erdős-Rényi or Chung-Lu graphs, and complex real-world networks that describe the interactions in the biological invasion process. These networks, while being significantly more complex than standard models, are still amenable to theoretical and experimental analyses as we demonstrate in this work.

#### 1.2.2. Theoretical Results on Spectral Radius and Diameter

We derive bounds on the spectral radius and diameter of MPSN model. These two graph invariants are important indicators of the diffusion dynamics. The bounds highlight the importance of inter-locality and intra-locality components of the network, which represent the human factors in the spread.

#### 1.2.3. Experimental Analysis of Synthetic and Real-World Networks

We report results from extensive experiments conducted on hundreds of synthetic networks using the MPSN model and several real-world networks. First, we study how the MPSN model parameters influence the spectral radius and diameter. This is followed by running simulations for varying pathway probabilities. In both analyses, to understand the influence of model parameters (i.e., parameters of the network and of the diffusion process) on the extent of spread, we view it as a supervised learning problem. Here the feature vector is comprised of model parameters and the observed variable is a graph invariant (in the structural analysis) or a simulation output (in the dynamical analysis), and we use regression trees and random forests to identify the primary drivers of diffusion process. Our experimental analyses help to identify the regimes where long distance edges and locality size are highly influential in increasing the spread.

## 2. Related work

### 2.1. Networked Representations of Invasive Species Spread

The current state-of-the-art for modeling invasive species involves developing risk maps using ecological niche models (Venette et al., 2010). Such models account for climate and biology of the invasive species and its hosts to map the long-term establishment potential. They do not provide a causal explanation to the extent and dispersion of spread or explicitly account for human-mediated pathways. However, in recent years, network diffusion models are being increasingly applied to model the spread dynamics of invasive species in order to account for their long distance spread. Hernandez Nopsa et al. (2015) studied the structure of rail networks for grain transport in the United States and Eastern Australia to identify the shortest paths for the anthropogenic dispersal of pests and mycotoxins, as well as the major sources, sinks, and bridges for movement. Sutrave et al. (2012) used an SI model (Easley and Kleinberg, 2010) to county-to-county network to model wind speed and direction, and host density to identify locations to monitor soybean rust, a pathogen. Koch et al. (2014) assess the risk of forest pests due to camping activities. Venkatramanan et al. (2020) used a Bayesian inference method to identify the most likely spread pattern of an invasive pest by modeling the spread as a diffusion process on a time varying network.

### 2.2. Complex Multi-Pathway Dispersal Models

Carrasco et al. (2010) considered both local and long distance spread in a process-based spatially explicit simulation model to study pest of the maize crop. They use phenology models to estimate pest population size, a negative power law kernel for self-mediated spread, and a gravity model representation of long distance edges. Our work is based on a similar approach by McNitt et al. (2019) who model the multi-pathway spread by accounting for self-mediated spread and spread within and between areas of high human activity (e.g., urban areas). Both works account for suitability of establishment and the distribution of host crop production. Similar modeling approaches have been applied to study infectious diseases in humans and livestock (Ajelli et al., 2010; Kim et al., 2018; Venkatramanan et al., 2021).

### 2.3. Spectral Characterization of Network Dynamics

Several structural properties of networks have been used to understand the progression of a diffusion process. These include basic properties such as degree distribution or clustering coefficient to other properties such as graph spectrum, diameter, and degeneracy, to name a few. The **spectrum** of a graph is the set of eigenvalues of its adjacency matrix. There are several works that relate spectrum, particularly the first eigenvalue or *spectral radius* λ_1_(*G*) of the adjacency matrix of a graph *G*, to disease spread in SEIR-like epidemic models (Ganesh et al., 2005; Prakash et al., 2012). A well known result that highlights the impact of the network structure on the dynamics is the following: an epidemic dies out “quickly” if λ_1_(*G*) ≤ *T*, where *T* is a threshold that depends on the disease model. This relationship has motivated a number of works on epidemic control where the objective is to find an optimal set of nodes (or edges) to remove from the network that leads to the maximum reduction in its spectral radius (Van Mieghem et al., 2011; Saha et al., 2015; Zhang et al., 2016; Chen et al., 2021).

### 2.4. Diameter and Network Dynamics

The diameter of real-world networks is an important structural parameter used to characterize epidemics on real-world graphs (Holme, 2013; Pastor-Satorras et al., 2015). In the literature, average path lengths between pairs of nodes and diameter of the network have been observed to have an effect on the rate of diffusion. In particular, the lower the diameter, the higher is the diffusion rate (Banos et al., 2015; Taghvaei et al., 2020; Kamra et al., 2021). In spatial networks, the diameter tends to be large when compared to social networks that exhibit the small-world effect (Watts and Strogatz, 1998), and long distance edges can be responsible for bringing the diameter down (Barthélemy, 2011).

### 2.5. Machine Learning and Simulation Systems

From the works described above, it is clear that even simple SIR-like processes (Easley and Kleinberg, 2010) on arbitrary static networks are difficult to characterize. In recent years, there have been several studies that use a combination of extensive simulations and machine learning algorithms to understand the phase space of complex models. Fox et al. (2019) explored multiple ways in which such a nexus of the two modeling approaches can be used to understand complex systems. Lamperti et al. (2018) used extreme gradient boosted trees for the purpose of phase space exploration of a complex model. Our approach to apply classification and regression trees (CART) and random forests (Breiman, 2001, 2017) is motivated by this work. Other methods based on Gaussian emulators have been used for calibrating agent-based models (Fadikar et al., 2018).

## 3. Preliminaries

### 3.1. Graph Theoretic Concepts

We begin with the definitions of several basic graph theoretic concepts. Additional information regarding these concepts can be found in West (2006); Brouwer and Haemers (2012). We assume that graphs are simple (i.e., they have no multi-edges or self loops).

Given an undirected graph *G*(*V, E*), where *V* = {*v*_1_, *v*_2_, …, *v*_*n*_}, the **adjacency matrix** *A*_*G*_ is an *n* × *n* (symmetric) matrix defined as follows.


$$A_{G}\left[i,j\right]=\{\begin{array}{cc} 0 & \text{if }i=j\\ 1 & \text{if }v_{i}\;\text{and }v_{j}\;\text{are adjacent and}\\ 0 & \text{otherwise}. \end{array}$$

We use degree(*v*_*i*_) to denote the degree of node *v*_*i*_. Also, we use δ(*G*) and Δ(*G*), respectively, to denote the minimum and maximum node degrees in *G*.

Consider a connected undirected graph *G*(*V, E*) with a non-negative weight *w*(*e*) on each edge *e* ∈ *E*. When *w*(*e*) = 1 for each *e* ∈ *E*, we sometimes refer to *G* as an *unweighted* graph. A **shortest path** between any pair of nodes *v*_*i*_ and *v*_*j*_ is a path such that the total weight of all the edges in the path is a minimum among all the paths between *v*_*i*_ and *v*_*j*_. Let *d*_*G*_(*v*_*i*_, *v*_*j*_) denote the length of a shortest path between *v*_*i*_ and *v*_*j*_. Then, the **diameter** of *G* is defined by


$$\mathrm{diam}\left(G\right)\;=\max\left\{d_{G}\left(v_{i},v_{j}\right)\;:v_{i},v_{j}\in V\right\}.$$

If *G* is not connected, then by convention, diam(*G*) is taken as ∞.

Given an undirected graph *G*(*V, E*) and an integer *t* ≥ 1, the **t-th** power of *G*, denoted by *G*^*t*^, is an undirected graph *G*^*t*^(*V, E*^*t*^), where $\left\{v_{i},v_{j}\right\}\in E^{t}$ iff there is a path with at most *t* edges between *v*_*i*_ and *v*_*j*_ in *G*. Given two graphs *G*_1_(*V*_1_, *E*_1_) and *G*_2_(*V*_2_, *E*_2_), the **Kronecker product** of *G*_1_ and *G*_2_, denoted by *G*_1_ × *G*_2_, is the graph *G*′(*V*′, *E*′), where $V^{\prime }=V_{1}\times V_{2}$ and an edge {(*a, b*), (*c, d*)} is in *E*′ iff {*a, c*} ∈ *E*_1_ and {*b, d*} ∈ *E*_2_.

### 3.2. Matrix Concepts

For any *n* × *n* symmetric matrix *M* with real entries, it is known that all the *n* eigen values, denoted by λ_1_, λ_2_, …, λ_*n*_, are real (Brouwer and Haemers, 2012). Since the *n* × *n* adjacency matrix of an undirected graph with *n* nodes is symmetric and all its entries are from {0,1}, it follows that all the eigen values of the adjacency matrix are real. We will assume without loss of generality that these *n* eigen values are ordered so that λ_1_ ≥ λ_2_ ≥ … ≥ λ_*n*_. Thus, λ_1_ will be referred to as the **first** eigen value or the **spectral radius**.

### 3.3. Geometric Concepts

For any pair of points *a* = 〈*a*_*x*_, *a*_*y*_〉 and *b* = 〈*b*_*x*_, *b*_*y*_〉 in the 2D-plane, let $d_{\text{Euc}}\left(a,b\right)=\sqrt{{\left(a_{x}-b_{x}\right)}^{2}+{\left(a_{y}-b_{y}\right)}^{2}}$ denote the Euclidean distance between *a* and *b*. It is well known that the Euclidean distance satisfies the **triangle inequality**: for any three points *a*, *b*, and *c*, *d*_Euc_(*a, b*) + *d*_Euc_(*b, c*) ≥ *d*_Euc_(*a, c*). As a generalization of this inequality, we have the following fact: if *a*_1_, *a*_2_, …, *a*_*n*_ are *n* ≥ 3 points in the plane, then $\sum_{i=1}^{n-1}d_{\text{Euc}}\left(a_{i},a_{i+1}\right)$ ≥ *d*_Euc_(*a*_1_, *a*_*n*_). We will use this inequality in Section 6.

## 4. Multi-Pathway Spatial Network

### 4.1. Motivation

A number of models have been proposed for spatial networks, where nodes and edges are embedded in a *d*-dimensional space with some geometric constraints. Such a network model acts as a reference model for comparison with real-world networks, and is amenable to theoretical analysis and controlled experimentation. Network models are also a principled approach to approximate partially-known real-world networks and to capture their variability (Gutfraind et al., 2015). Barthélemy (2011) provides a comprehensive review of such models. Lattice graphs and random geometric graphs are examples of some of the simplest spatial networks. More complex models that exhibit properties found in real-world spatial networks have also been proposed. Most of these models have been derived by spatial generalizations of well-known random graph models, such as Erdős-Rényi graphs, the Watts-Strogatz model, and the preferential attachment model Easley and Kleinberg (2010). However, to the best of our knowledge, none of these models is representative of networks arising out of biological spread processes like epidemics of invasive species or infectious diseases. While these network models have a base component that is grid- or lattice-like, they are missing a multi-scale component due to human-mediated pathways of spread. At the same time, the domain specific studies are limited to simulations on a single instance of the network that is usually constructed using a combination of available data and spatial interaction models such as the gravity model. Uncertainty quantification and sensitivity analyses are typically limited to diffusion model parameters, not network structure. Here, we address these gaps by developing a model that is a realistic representation of such processes, and analyzing its structural parameters such as spectral radius and diameter that are well-studied in the context of diffusion processes on networks. Later, in Sections 5 and 6, we will further discuss how the network model parameters affect these structural properties.

### 4.2. General Structure

At a high level, some aspects of this spatial network are captured in Figure 1. Here, we will describe the graph class *G*(*V, E*) of multi-pathway networks where *V* is a set of *n* vertices embedded in some suitable, 2-dimensional space *M* (e.g., Euclidean space ℝ^2^, the sphere *S*^2^). The graph *G* = *G*(*V, E*) is composed of three graphs *G*_*S*_, *G*_*L*_, and *G*_*LD*_, where the subscripts *S*, *L*, and *LD* denote corresponding pathways. With respect to Figure 1, *G*_*S*_ corresponds to the self-mediated spread, *G*_*L*_ is the spread within a locality, and *G*_*LD*_ is the inter-locality spread or long distance jumps. In general, *G* will be a loop-free, edge-labeled, and directed multi-graph. Edges are labeled by the pathway to which they belong with (*u, v*, π) encoding the edge from *u* to *v* due to pathway π. In the case where *G* is undirected we will use the notation ({*u, v*, }, π) for undirected edges, or simply {*u, v*} when the pathway is clear from the context. Let *r* denote the dispersal range. The graph *G*_*S*_ has vertex set *V* and an edge between each pair of vertices *v, v*′ ∈ *V* for which *d*(*v, v*′) ≤ *r* where *d* is some suitable metric on the embedding space *M*. Next, let $L=\left\{L_{1},L_{2},\ldots \right\}$ denote a collection of *n*_*L*_ mutually disjoint subsets of *V*. For each $L_{i}\in L$, let *E*_*i*_ be some edge set over *L*_*i*_, and let *G*_*i*_(*L*_*i*_, *E*_*i*_) denote the corresponding *locality graph*. The graph *G*_*L*_ = *G*_*L*_(*V*_*L*_, *E*_*L*_) is the (disjoint) union of the graphs *G*_*i*_. Let *F*_*LD*_ be a graph with vertex set L. We use a mapping *f* to define the edge set *E*_*LD*_ of *G*_*LD*_ via *F*_*LD*_ by associating to each edge (ℓ, ℓ′) of *F*_*LD*_ a set of edges $E_{f}\left(\ell ,\ell^{\prime }\right)\subset V\times V$ subject to the constraint that whenever (*v, v*′) ∈ *E*(ℓ, ℓ′) we have *v* ∈ ℓ and *v*′ ∈ ℓ′. The graph *G*_*LD*_ has vertex set *V* and its edge set is the union of the sets $E_{f}\left(\ell ,\ell^{\prime }\right)$ over all edges (ℓ, ℓ′) of *F*_*LD*_.

### 4.3. A Multi-Pathway Spatial Network Model

Here, we define the graph model class called *multi-pathway spatial network* (MPSN). Its vertex set consists of points on a $\sqrt{n}\times \sqrt{n}$ square lattice with integer coordinates (*i, j*), where $i,j\in \left\{0,1,2,\ldots ,\sqrt{n}-1\right\}$. We will assume throughout that *n* is a perfect square. Let *k* be a positive integer satisfying the following conditions: (i) it is a perfect square and (ii) $\sqrt{k}$ is a factor of $\sqrt{n}$. The vertex set is partitioned into *k* *regions*, each inducing a $\sqrt{n/k}\times \sqrt{n/k}$ subgrid. Each region consists of one *locality* with *s* vertices, where *s* has the same parity (odd or even) as *n*/*k*. Each locality is again a square grid of size $\sqrt{s}\times \sqrt{s}$ at the center of that region. For a locality *v*, let *L*_*v*_ denote the set of points on its square grid. Let L denote the set of localities.

As described above, we have three component graphs *G*_*S*_, *G*_*L*_, and *G*_*LD*_. The edge set of *G*_*S*_ is determined by the *range* parameter *r*. Any two lattice points are adjacent in *G*_*S*_ if they are at a *Euclidean distance* of at most *r*. The edge set of *G*_*L*_ is determined by a template graph *H*_*L*_, referred to as the *intra-locality graph*, on a $\sqrt{s}\times \sqrt{s}$ grid. For each locality *v*, let *G*_*v*_ denote the graph on the vertex set *L*_*v*_. Consider the bijection between the vertex sets of *G*_*v*_ and *H*_*L*_ induced by the natural ordering of their vertices on the $\sqrt{s}\times \sqrt{s}$ grid. Each *G*_*v*_ is isomorphic to *H*_*L*_ under this bijection. The graph *G*_*L*_ is the union of all graphs *G*_*v*_. Let *F*_*LD*_ be a graph defined on the localities, referred to as the *inter-locality graph*. The long distance human-mediated pathway graph *G*_*LD*_ is defined as follows: (*a, b*) ∈ *E*(*G*_*LD*_) ⇔ *a* ∈ *L*_*u*_, *b* ∈ *L*_*v*_ and (*u*, *v*) ∈ *E*(*F*_*LD*_).

We will henceforth denote this model by MPSN(*n, r, k, s, H*_*L*_, *F*_*LD*_). In the following discussion, we will consider scenarios where *F*_*LD*_ is sampled from some random graph model denoted by G. Examples of such models are Erdős-Rényi model and the preferential attachment model (Easley and Kleinberg, 2010). In such cases, *F*_*LD*_ is replaced by G, and the resulting notation is $\mathrm{MPSN}\left(n,r,k,s,H_{L},G\right)$.

#### 4.3.1. A Note on Directed Graphs

For simplicity, we only consider the undirected version of the multi-pathway network model. However, in real-world applications, these are typically directed weighted graphs that do not exhibit symmetry with respect to edge weights [see for example the networks in McNitt et al. (2019) or Carrasco et al. (2010)]. A natural directed version of the MPSN would correspond to *G*_*S*_ and *G*_*L*_ graphs with every undirected edge replaced by a pair of bidirectional edges and *F*_*LD*_ being a directed graph. Note that the resulting graph will be strongly connected.

## 5. Spectral Characterization

Here, we provide bounds for the spectral radius of an MPSN instance in terms of the spectral radii of the component networks representing the different pathways. The main objective is to understand the importance of each pathway in a diffusion process through the lens of graph spectra. Our main result is Theorem 5.1, where lower and upper bounds on the spectral radius of the multi-pathway network are expressed in terms of the parameters of the MPSN model.

**Theorem 5.1**. *Let G* = MPSN(*n, r, k, s, H*_*L*_, *F*_*LD*_) *be a multi-pathway spatial network. The spectral radius of G can be bounded as follows*:


$$\begin{array}{c} \max\left\{\frac{r^{2}}{2},\lambda_{1}\left(H_{L}\right),\lambda_{1}\left(F_{LD}\right)\left(s-1\right)\right\}\;\leq \;\lambda_{1}\left(G\right)\;\leq \;4r^{2}+\lambda_{1}\left(H_{L}\right)+\\ \lambda_{1}\left(F_{LD}\right)\left(s-1\right) \end{array}$$

The first term of both bounds in the above result corresponds to self-mediated spread (*G*_*S*_). We note that this is fully determined by the range parameter *r* and not by the size of the graph. Also, it increases as the square of the range. From an invasive species perspective, this suggests that a species with strong flying capability can rapidly expand its range. An example of such a recent global invasion is that of the fall armyworm (Westbrook et al., 2016; Day et al., 2017), which is known for its long distance migration. The second term in the bounds corresponds to intra-locality spread. This depends on the size and structure of the localities. A more significant contribution comes from the third term corresponding to the inter-locality spread pathway. It is proportional to the spectral radius of the inter-locality graph *F*_*LD*_. The denser the graph, the greater its contribution. More importantly, it is amplified by (*s*−1), the size of the locality. The main implication of this is that the higher the number of areas are that are suitable for establishment of the pest in a locality, the greater the influence of the inter-locality pathway.

The rest of the section is devoted to a proof of the above theorem. Throughout these proofs, we will denote the edge set of a graph *G* by *E*(*G*).

**Lemma 5.2**. *The spectral radius of the short-distance pathway network G*_*S*_ *with n nodes and range r is* Θ(*r*^2^).

*Proof*. Note that *G*_*S*_ is a network induced on a set of points located on an $\sqrt{n}\times \sqrt{n}$ grid with integer coordinates and range parameter *r*. We use the notation *u* = 〈*i, j*〉 for each vertex, where $i,j\in \left\{0,\ldots ,\sqrt{n}-1\right\}$ are the coordinates. Let *H*_1_ denote the grid graph where each 〈*i, j*〉 is adjacent to 〈*i* ± 1, *j*〉 and 〈*i, j* ± 1〉. Let *H*_2_ denote the graph where each 〈*i, j*〉 is adjacent to 〈*i* ± 1, *j*〉, 〈*i, j* ± 1〉, and 〈*i* ± 1, *j* ± 1〉. In the definitions of both *H*_1_ and *H*_2_, edges to vertices whose coordinates do not exist within the $\sqrt{n}\times \sqrt{n}$ grid are not added.

**Claim 1:** $E\left(H_{1}^{\left\lfloor r\right\rfloor }\right)\subseteq E\left(G_{S}\right)\subseteq E\left(H_{2}^{\left\lfloor r\right\rfloor }\right)$, where $H_{1}^{\left\lfloor r\right\rfloor }$ and $H_{2}^{\left\lfloor r\right\rfloor }$ are the ⌊*r*⌋th powers of *H*_1_ and *H*_2_, respectively.

**Proof of Claim 1:** We recall that *d*_*H*_(*u, v*) is the distance between *u* and *v* in graph *H* and *d*_Euc_(*u, v*) is the Euclidean distance between them. First, we will show that for any $\left(u,v\right)\in E\left(H_{1}^{\left\lfloor r\right\rfloor }\right)$, *d*_Euc_(*u, v*) ≤ *r*. To this end, it is enough to prove this for *u* = 〈0, 0〉 and *v* = 〈*x, y*〉 since both distances are shift invariant. For this case, *d*_Euc_(*u, v*)^2^ = *x*^2^ + *y*^2^. Since *u* and *v* are adjacent in $H_{1}^{\left\lfloor r\right\rfloor }$, *v* is reachable from *u* in at most ⌊*r*⌋ hops in *H*_1_. This means that *x* + *y* ≤ *r*, with *x* and *y* being positive. Therefore, *x*^2^ + *y*^2^ ≤ *x*^2^ + (*r* − *x*)^2^ ≤ *r*^2^ + 2*x*(*x*−*r*) ≤ *r*^2^, and $E\left(H_{1}^{\left\lfloor r\right\rfloor }\right)\subseteq E\left(G_{S}\right)$. Now, we will show that if *x*^2^ + *y*^2^ ≤ *r*^2^, then $\left(u,v\right)\in E\left(H_{2}^{\left\lfloor r\right\rfloor }\right)$. Since *x*^2^ + *y*^2^ ≤ *r*^2^ and *x* and *y* are integers, we have *x, y* ≤ ⌊*r*⌋. Note that in *H*_2_, *d*_*H*_2__(〈0, 0〉, 〈*x, y*〉) = max(*x, y*) ≤ ⌊*r*⌋. This is because, assuming without loss of generality, that *x* ≤ *y*, 〈*x, y*〉 is reachable from 〈0, 0〉 by the path 〈0, 0〉〈1, 1〉⋯〈*x, x*〉〈*x, x* + 1〉⋯〈*x, y*〉, hence $E\left(G_{S}\right)\subseteq E\left(H_{2}^{\left\lfloor r\right\rfloor }\right)$, completing our proof of Claim 1.

We now recall a result from spectral graph theory (Brouwer and Haemers, 2012).

**Lemma 5.3 (Brouwer and Haemers, 2012)**. *For any undirected graph G*, δ(*G*) ≤ λ_1_(*G*) ≤ Δ(*G*), *where* δ(*G*) *and* Δ(*G*) *denote the minimum and maximum node degree in G*.

**Claim 2:** (i) $\delta \left(H_{1}^{\left\lfloor r\right\rfloor }\right)\geq r\left(\left\lfloor r\right\rfloor +1\right)/2$. (ii) $\Delta \left(H_{2}^{\left\lfloor r\right\rfloor }\right)\leq 4{\left(\left\lfloor r\right\rfloor -1\right)}^{2}+4\left(\left\lfloor r\right\rfloor \right)$.

**Proof of Claim 2:** To prove Part (i), note that in $H_{1}^{\left\lfloor r\right\rfloor }$, the nodes with minimum degree are the corner vertices of the grid. Recalling that 〈0, 0〉 is a corner vertex, and any neighbor 〈*x, y*〉 satisfies *x* + *y* ≤ ⌊*r*⌋, its degree is ⌊*r*⌋(⌊*r*⌋ + 1)/2.

To prove Part (ii), note that in $H_{2}^{\left\lfloor r\right\rfloor }$, if max(*x, y*) ≤ ⌊*r*⌋, then 〈*x, y*〉 is a neighbor of 〈0, 0〉. There are ⌊*r*⌋^2^−1 such vertices. For sufficiently large *n* in comparison to *r*, a non-corner vertex can have a degree of up to 4(⌊*r*⌋ − 1)^2^ + 4(⌊*r*⌋). This completes our proof of Claim 2.

We now continue with our proof of Lemma 5.2. As argued earlier, $E\left(H_{1}^{\left\lfloor r\right\rfloor }\right)\subseteq E\left(G_{S}\right)\subseteq E\left(H_{2}^{\left\lfloor r\right\rfloor }\right)$. Thus, $\delta \left(H_{1}^{\left\lfloor r\right\rfloor }\right)\leq \delta \left(G_{S}\right)$ and so from Lemma 5.3 and Part (i) of Claim 2, we have λ_1_(*G*_*S*_) ≥ δ(*G*_*S*_) ≥ ⌊*r*⌋(⌊*r*⌋ + 1)/2 = Ω(*r*^2^). Likewise, $\Delta \left(G_{S}\right)\leq \Delta \left(H_{2}^{\left\lfloor r\right\rfloor }\right)$. So from Lemma 5.3 and Part (ii) of Claim 2, we have $\lambda_{1}\left(G_{S}\right)\leq \Delta \left(G_{S}\right)\leq 4{\left(\left\lfloor r\right\rfloor -1\right)}^{2}+4\left(\left\lfloor r\right\rfloor \right)$ = *O*(*r*^2^). We thus conclude that $\lambda_{1}\left(G\right)=\Theta \left(r^{2}\right)$.     □

**Lemma 5.4**. *Consider the local human-mediated spread pathway network G*_*L*_ *with n nodes, number of regions k and locality size s. Let H*_*L*_ *be the intra-locality graph. The set of eigenvalues of the adjacency matrix G*_*L*_ *is identical to the set of eigenvalues of the adjacency matrix of H*_*L*_.

*Proof*. By design, *G*_*L*_ induces multiple connected components, with each component being isomorphic to *H*_*L*_. The result follows.     □

**Lemma 5.5**. *Consider the local human-mediated spread pathway network G*_*LD*_ *with n nodes, number of regions k and locality size s. Let F*_*LD*_ *be the inter-locality graph. The spectral radius of* *G*_*LD*_ *equals* (*s* − 1)λ_1_(*F*_*LD*_).

*Proof*. We will use the definition of localities from Section 4.3 and the definition of Kronecker product of graphs from Section 3.

By definition, (*a, b*) ∈ *E*(*G*_*LD*_) ⇔ *a* ∈ *L*_*u*_, *b* ∈ *L*_*v*_ and (*u*, *v*) ∈ *E*(*F*_*LD*_). Also, each *L*_*u*_ is of the same size *s*. Let *G*_*K*_ denote a graph on *s* vertices which induces a complete graph. Now we show that the graph *G*_*LD*_ is equivalent to the graph *H* obtained as the Kronecker product of *F*_*LD*_ and *G*_*K*_. Since *G*_*K*_ has *s* nodes, we can define a bijection between *V*(*G*_*K*_) and *V*(*L*) for any locality *L*. Let this bijection be denoted by π_*u*_:*V*(*G*_*K*_) → *L*_*u*_. Let (*u*, *x*), (*v*, *y*) ∈ *V*(*H*) where *u*, *v* ∈ *V*(*F*_*LD*_) and *x, y* ∈ *V*(*G*_*K*_).

Suppose (*u*, *x*) and (*v*, *y*) are two vertices in *H*, where *u* ≠ *v*, *a* = π_*u*_(*x*) and *b* = π_*v*_(*y*). We will now show that {(*u*, *x*), (*v*, *y*)} is an edge in *H* iff *a* is adjacent to *b* in *G*_*LD*_. Suppose {(*u*, *x*), (*v*, *y*)} is an edge in *H*. Since (*u*, *x*) is adjacent to (*v*, *y*), we have that *u* is adjacent to *v* in *F*_*LD*_ and *x* is adjacent to *y* in *G*_*K*_. Note that since *G*_*K*_ is a complete graph, the latter condition will always be true. Since *a* belongs to locality *u* and *b* belongs to locality *v*, it follows by the definition of *G*_*LD*_ that *a* and *b* are adjacent as well. Now suppose {*a, b*} ∈ *G*_*LD*_. Since the two vertices belong to different localities, they can be adjacent only because *u* is adjacent to *v* in *F*_*LD*_. Again noting that *G*_*K*_ is a clique, $x=\pi_{\underset{\_}{u}}^{-1}$ and $y=\pi_{\underset{\_}{v}}^{-1}$ are adjacent in *G*_*K*_. Therefore, {(*u*, *x*), (*v*, *y*)} is an edge in *H*.

Since *G*_*K*_ is a clique, its spectral radius is *s* − 1 (Brouwer and Haemers, 2012). By above arguments, λ_1_(*H*) = λ_1_(*G*_*LD*_). Since *H* is a Kronecker product of *F*_*LD*_ and *G*_*K*_, its spectral radius is λ_1_(*F*_*LD*_)λ_1_(*G*_*K*_) (Brouwer and Haemers, 2012). Since λ(*G*_*K*_) = *s* − 1, it follows that λ_1_(*H*) = (*s* − 1)λ_1_(*F*_*LD*_). This completes our proof of Lemma 5.5.     □

From Lemmata 5.2, 5.4, and 5.5, the proof of Theorem 5.1 follows by (i) noting that the spectral radius of any graph is at least as large as the spectral radius of any of its subgraphs, and (ii) for any two Hermitian matrices *A* and *B* (such as the adjacency matrices of graphs), λ_1_(*A* + *B*) ≤ λ_1_(*A*) + λ_1_(*B*).

## 6. Diameter

In this section, we derive an upper bound on the diameter of an MPSN instance, where the inter-locality graph is drawn from an Erdős-Rényi model. In particular, we show that the addition of a few long distance edges corresponding to the inter-locality graph can lead to a significant decrease in the diameter. Our main result is as follows.

**Theorem 6.1**. *Let* $G\in \mathrm{MPSN}\left(n,r,k,s,H_{L},G\right)$ *be an instance of the multi-pathway network model where* $F_{LD}\in G$ *is an instance of the Erdős-Rényi random graph model* G=H(k,ϵ/k) *for some ϵ > 0. (i) The diameter of the graph G*_*S*_ *(i.e., G without the inter- and intra-locality edges) is* $\Omega \left(\sqrt{n}/r\right)$. *(ii) The diameter of G is asymptotically almost surely* $O\left(\sqrt{\frac{n}{k}}\log k\right)$.

This theorem implies that, for a sufficiently large *k* (number of localities), even when the range *r* is low, a small number of long distance edges can reduce the diameter by a significant amount. In practice, this reduction in diameter is significant since the value of *r* is small while *k* is a much larger integer. From an application perspective, this suggests that even organisms with limited flying ability can cover great distances in a few hops due to long distance trade or travel links.

To prove Theorem 6.1, we use the following result on the diameter of randomly perturbed graphs due to Krivelevich, Reichman and Samotij (Krivelevich et al., 2015). Their main observation is that if for some small ϵ > 0, approximately ϵ*n* random edges are added to any *n*-node graph, then asymptotically almost surely, the diameter of the resulting graph is *O*(log*n*). Formally, their result is stated as follows.

**Lemma 6.2 (Krivelevich, Reichman and Samotij (Krivelevich et al., 2015))**. *For every ϵ* > 0, *there exists C* > 0 *such that the following holds. Let G be an n-vertex connected graph, choose* $R\sim G\left(n,\frac{\epsilon }{n}\right)$ and let G^*^ = *G*∪*R*. Then, asymptotically almost surely, the diameter of G^*^ *is at most C* log *n*.

*Proof of Theorem 6.1:* **Part (i):** First, we will prove the lower bound on the diameter of *G*_*S*_. On the grid over which *G*_*S*_ is defined, consider the two nodes *x* = 〈0, 0〉 and $y=\langle \sqrt{n}-1,\sqrt{n}-1\rangle$. The Euclidean distance between these two nodes is *d*_Euc_(*x, y*) = $\sqrt{2\left(n-2\sqrt{n}+2\right)}$. It can be verified that for *n* ≥ 9, $d_{\text{Euc}}\left(x,y\right)\geq \sqrt{n}$. Suppose the length of a shortest path *P* between *x* and *y* in *G*_*S*_ has ξ edges. By the definition of *G*_*S*_, each of these edges has a geometric length of at most *r*. Therefore, the total geometric length of all these edges in *P* is at most *rξ*. Thus, by the generalized triangle inequality (Section 3), the distance between *x* and *y* is at most *rξ*. As noted above, for *n* ≥ 9, this distance is at least $\sqrt{n}$. Thus, $r\xi \geq \sqrt{n}$ or $\xi \geq \frac{\sqrt{n}}{r}$ = $\Omega \left(\sqrt{n}/r\right)$. In other words, there is at least one pair of nodes in *G*_*S*_ for which the shortest path uses $\Omega \left(\sqrt{n}/r\right)$ edges. Therefore, the diameter of *G* is $\Omega \left(\sqrt{n}/r\right)$, and this completes our proof of Part (i).

**Figure 2:**
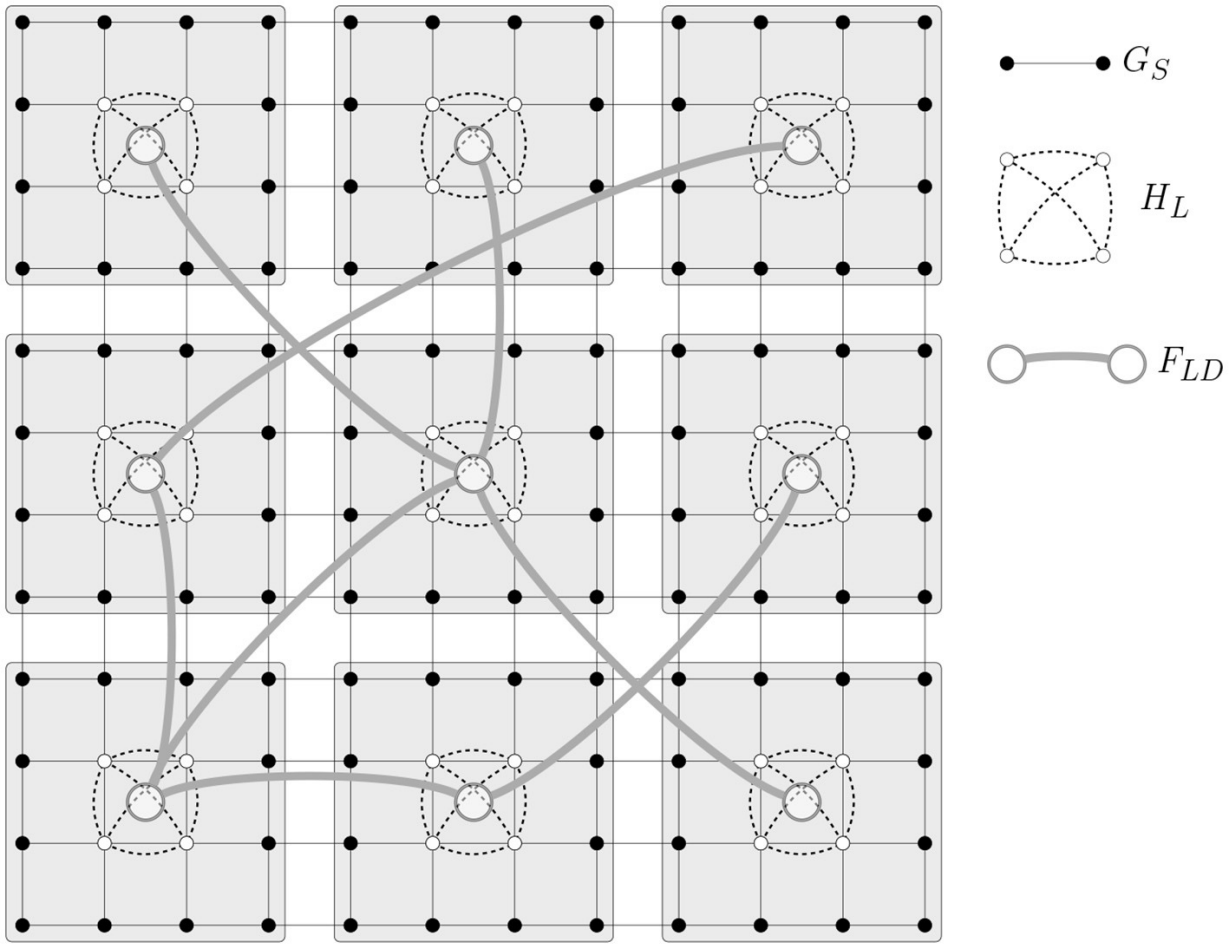
An instance of the multi-pathway spatial network model MPSN(*n, r, k, s, H*_*L*_, *F*_*LD*_). Here, number of nodes *n* = 144, number of regions *k* = 9, and locality size *s* = 4. The range *r* = 1 resulting in a grid graph for *G*_*S*_. Here, the intra-locality graph *H*_*L*_ is a clique on four vertices.

**Part (ii):** Recall that L is the set of localities. By definition, there are *k* localities placed on a $\sqrt{k}\times \sqrt{k}$ grid (see Figure 2). We will first create a base graph *F*_*B*_ on L by adding edges between neighbors on the grid. More formally, we will assume that the coordinates of each locality 〈*x, y*〉 satisfy $x,y\in \left\{0,1,2,\ldots ,\sqrt{k}-1\right\}$. In *F*_*B*_, a locality with coordinates 〈*x, y*〉 is adjacent to 〈*x* ± 1, *y* ± 1〉 (if nodes with those coordinates are in the same locality). Noting that *F*_*B*_ is connected and by assumption, *F*_*LD*_ ∈ *H*(*k*, ϵ/*k*), from Lemma 6.2, it follows that the diameter of the graph *F*_*LD*_ ∪ *F*_*B*_ obtained by adding the edges of *F*_*LD*_ to *F*_*B*_ is asymptotically almost surely at most *c*log*k* for some positive constant *c*.

Now, we will prove the upper bound on the diameter of *G*. Let *u, v* ∈ *V* be two distinct nodes on the grid. Let *L*_*u*_ and *L*_*v*_ be the localities closest to *u* and *v* respectively. The distance from *u* to the closest vertex $u^{\prime }\in L_{\underset{\_}{u}}$ is at most most $2\left(\sqrt{n/k}-s\right)/\left\lceil \frac{r}{\sqrt{2}}\right\rceil$. The same holds for *v*. As proven above, there exists a path *P* of length at most *c*log*k* almost surely from *u* to *v* in *F*_*LD*_∪*F*_*B*_. Let *P* = *u* − *u*_1_ − *u*_2_ − ⋯ − *u*_*t*_ − *v*. Some of the edges in this path belong to *F*_*B*_ and the rest to *F*_*LD*_. If *u*_*i*_*u*_*i*+1_ belongs to *F*_*LD*_, then by definition of *G*_*LD*_, there exist corresponding nodes *u*_*i*_ ∈ *L*_*u*_*i*__ and *u*_*i*+1_ ∈ *L*_*u*_*i*+1__ such that *u*_1_ and *u*_2_ are adjacent in *G*_*LD*_ and, therefore, are adjacent in *G*. If on the other hand, *u*_*i*_ and *u*_*i*+1_ are adjacent in *F*_*B*_, then, any two vertices *u*_*i*_ ∈ *L*_*u*_*i*__ *u*_*i*+1_ ∈ *L*_*u*_*i*+1__ are at a distance at most $2\left(\sqrt{n/k}-s\right)/\left\lceil \frac{r}{\sqrt{2}}\right\rceil +2\mathrm{diam}\left(G_{L}\right)$. The first term, which corresponds to inter-locality distance on the grid, is clearly $O\left(\sqrt{n/k}\right)$. The second term corresponds to node-to-node distance within a locality. Note that $\mathrm{diam}\left(G_{L}\right)\leq s/\left\lceil \frac{r}{\sqrt{2}}\right\rceil$. Noting that $s\leq \sqrt{k}$ and $k\leq \sqrt{n}$, it can be verified that $\mathrm{diam}\left(G_{L}\right)=O\left(\sqrt{n/k}\right)$. Thus, the length of each edge in *P* is $O\left(\sqrt{n/k}\right)$ and the number of edges in *P* is asymptotically almost surely at most *c*log*k*. It follows that the length of path *P* from *u* to *v* is asymptotically almost surely $O\left(\sqrt{\frac{n}{k}}\log k\right)$. This completes our proof of Part (ii).     □

## 7. A Multi-Pathway Diffusion Model

We now describe briefly the model that is based on the approach of McNitt et al. (2019), referred to as the SPREAD model. We refer to Figure 1 and Section 4 for the network representation on which the diffusion process is based. There are three pathways of spread. Self-mediated dispersal corresponds to diffusion from one node to its neighboring nodes, intra-locality dispersal is diffusion within a locality (farmer-market interactions), and inter-locality diffusion corresponds to long distance dispersal from one cell to another (trade). The diffusion model is a discrete-time SEI process where a node transitions from **E** to **I** after ℓ time steps. The transition from **S** to **E** is described below.

Each node *v* has a periodic time-varying attribute, namely its infectivity ρ(*v, t*). The probability that a node can be infected through a pathway is modeled as a negative exponential function of infectivity and pathway parameters, which can be expressed as edge weights between two nodes as follows. For the short-distance dispersal, the probability that node *v* is infected by any “neighbor” *v*′ that is at a distance at most *r* (range) is given by


(1) $$\begin{array}{l} w\left(v^{\prime },v,P_{s},t\right)\;=\;1-\exp\left(-\alpha s\rho \left(v^{\prime },t\right)\right), \end{array}$$

where *P*_*s*_ is the edge label corresponding to the pathway and α*s* is a tunable pathway parameter. For two nodes *v* and *v*′ within a locality, the probability of within intra-locality transmission from *v*′ to *v* is given by


(2) $$\begin{array}{l} w\left(v^{\prime },v,P_{\ell },t\right)\;=\;1-\exp\left(-\alpha \ell \rho \left(v^{\prime },t\right)\right), \end{array}$$

where *P*_ℓ_ is the pathway label and αℓ is the tunable pathway parameter. For the inter-locality transmission, the weights on the flow network *F*_*LD*_ influence the probability of transmission. Suppose *v* ∈ *L*_*v*_ and $v^{\prime }\in L_{\underset{\_}{v^{\prime }}}$; then the probability that *v* is infected by *v*′ through this pathway is given by


(3) $$\begin{array}{l} w\left(v^{\prime },v,P_{\ell d},t\right)\;=\;1-\exp\left(-\alpha \ell dF_{\underset{\_}{v^{\prime }}\underset{\_}{v}}\rho \left(v^{\prime },t\right)\right), \end{array}$$

where *P*_ℓ*d*_ is the pathway label and α*ℓd* is the tunable pathway parameter.

As mentioned earlier, the dynamics of the SPREAD model follow the Susceptible-Exposed-Infectious (SEI) (Marathe and Vullikanti, 2013), as defined below. Each node is in one of the following states: susceptible (**S**), exposed (**E**), or infectious (**I**). Let S ⊆ *V* denote the initial set of nodes in state **I**. The nodes in S serve as the seeds for the diffusion process. At any time *t*, each node *u* in state **I** infects its susceptible neighbor *v* with probability equal to the weight *w*(*u, v, P, t*) via the labeled edge (*u, v, P*). An exposed node (i.e., a node in state **E**) transitions to the infectious state after ℓ time steps, where ℓ is the latency period. It corresponds to the time taken for the node to transition from being exposed to being infectious.

## 8. Experiments

### 8.1. Outline

The extent and pattern of spread in a network depend on both the network structure and the diffusion model properties. Our goal here is to identify the drivers of the diffusion process. We will study how combinations of these properties affect the spread in the network. Specific studies are as follows.

1. We study how MPSN model parameters such as range, number of localities and the density of the intra- and inter-locality graphs affect the structure of the network from the perspective of dynamics. Specifically, we analyze the evolution of the spectral radius (λ_1_(*G*)) and diameter (diam(*G*)) with respect to model parameters.
2. We analyze the networks considered above with respect to the SPREAD diffusion model. Here, the objective is to study how combinations of the pathway probabilities determined by α*s*, αℓ, and α*ℓd* and network model parameters affect the spread. We also study how combinations of structural and dynamical properties influence the spread. Further, we investigate how representative network parameters (such as spectral radius and diameter) are in characterizing SEI-like diffusion processes especially when non-uniform edge probabilities are involved.
3. Based on the insights derived from the above two studies, we analyze the properties of several real-world commodity flow networks (McNitt et al., 2019). These networks are temporal and edge-weighted unlike MPSN considered in the two studies above. We analyze the structural properties of the temporal snapshots of these networks.

### 8.2. Networks and Experiment Design

All the code used to generate the results is made publicly available (Adiga, 2021). The parameters used to generate synthetic networks corresponding to the MPSN model and the characteristics of real-world datasets corresponding to domestic trade of tomatoes are provided in Tables 1, 2, respectively. For the synthetic networks, we considered a 64 × 64 grid of nodes. Using a full factorial design, networks were constructed for combinations of parameters in Table 1. For the inter-locality graph *F*_*LD*_, we used the Erdős-Rényi random graph model where the edge probabilities were chosen to be functions of the number of nodes of *F*_*LD*_, which in turn is equal to the number of regions *k*. However, not all combinations of parameters are valid. Since these networks have a random graph component, for each valid combination of parameters, we constructed 10 replicates. Thus, over 7,000 networks were constructed. We note that while the synthetic networks considered here are undirected and unweighted, the real-world networks considered (Table 2) are directed and edge-weighted. In addition, these temporal networks are also periodic: there are 12 snapshots of networks, one for every month of the year induced by the seasonal production and flow of commodity around the year. The details of network construction are given in McNitt et al. (2019).

**Table 1:** List of model parameters used in the experiments for the MPSN model.

**Parameters**	**Values**
Number of nodes *n*	4096
Number of regions *k*	4, 16, 64
Range *r*	1, 1.5, 2, …, 4
Locality size *s*	4, 16, 64
Intra-locality graph *H*_*L*_	Complete graph, star graph
Inter-locality graph *F*_*LD*_	*G*(*n, p*)
ϵ: (inter-locality graph edge probability ϵ/*k*)	0.1, 0.5, 1, 5, 10

**Table 2:** List of real-world networks used and their attributes.

**Net**.	**Name**	**#Nodes**	**#Edges**	**#Groups**	**#Group edges**
BD	Bangladesh	211	6846	7	141
ID	Indonesia	3296	110640	35	2181
PH	Philippines	673	20108	16	450
TH	Thailand	738	27666	5	48
VN	Vietnam	503	16746	15	426

*Each of these networks is constructed using various datasets on production, trade, and consumption of tomatoes (McNitt et al., 2019). These networks are edge-weighted*.

Structural properties were computed for all the synthetic networks. A subset of these networks was used for dynamical analysis using the diffusion model presented in Section 7. For this, a range of parameter values corresponding to the SPREAD diffusion model of Section 7 were used. This is listed in Table 3. The number of simulation instances in each case was 100. Since diffusion is modeled as an SEI process, assuming that the network is strongly connected, the fixed points or the equilibrium points of the system are either all nodes being in the infected state or all nodes being in the susceptible state. Therefore, we are interested in the state of the system at specific time steps. The observed variable is the mean number of nodes infected by a given *time horizon* or by *time step* *T*. For the initial seeding, five percent of the nodes were chosen uniformly at random and set to the infected state.

**Table 3:** List of network and SPREAD model parameters used in the experiments for the MPSN model.

**Parameters**	**Values**
Number of nodes *n*	4096
Number of regions *k*	16
Range *r*	1, 2, 3
Locality size *s*	4, 16
Intra-locality graph *H*_*L*_	Complete graph
Inter-locality graph *F*_*LD*_	*G*(*n, p*)
ϵ: (inter-locality graph edge probability ϵ/*k*)	0.1, 1, 10
α*s*: grid pathway parameter	0, 0.001, 0.005, 0.01, 0.015, 0.01
αℓ: intra-locality pathway parameter	0, 0.001, 0.005, 0.01, 0.015, 0.01
α*ℓd*: inter-locality pathway parameter	0, 0.001, 0.005, 0.01, 0.015, 0.01
ρ: Infectivity of a node	1 for all nodes
Edge weights	1 for all edges
ℓ: exposure delay	0
Time horizon *T*	6, 12, 18, 24

### 8.3. Structural Analysis of MPSN Model

**Figure 3:**
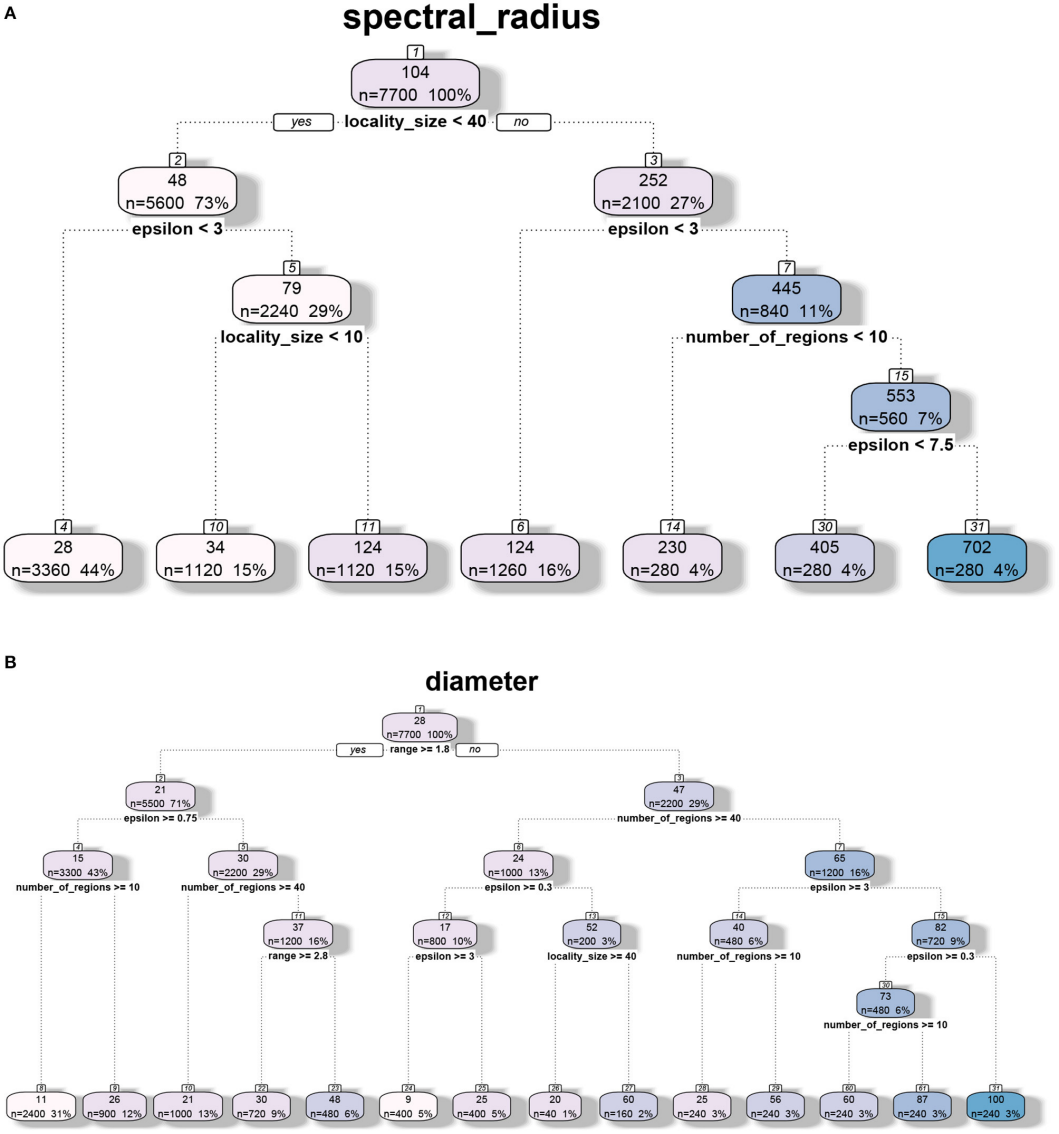
Regression tree analysis of network properties with respect to model parameters for the MPSN model. The properties were computed for various networks given in Table 1. **(A)** CART analysis: Spectral radius λ1(G). **(B)** CART analysis: Diameter diam (G).

**Figure 4:**
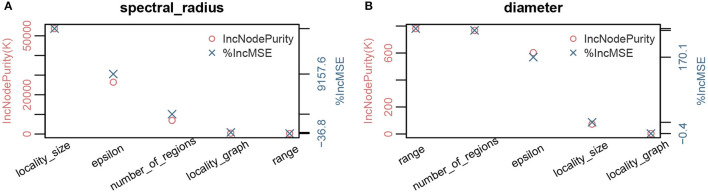
Parameter importance using random forest analysis of network properties with respect to model parameters for the MPSN model. The properties were computed for various networks given in Table 1. **(A)** Parameter sensitivity: Spectral radius λ1(G). **(B)** Parameter sensitivity: Diameter diam(G).

The decision tree analysis of spectral radius λ_1_(*G*) in Figures 3, 4 indicates that the primary drivers of its value are locality size *s* and the inter-locality edge probability factor ϵ. The significance of the former can be attributed to the model. We recall that the dominating term in the bounds provided in Theorem 5.1 is λ_1_(*G*_*LD*_) = λ_1_(*F*_*LD*_)(*s* − 1) consisting of the locality size and ϵ that influences λ_1_(*F*_*LD*_). For Erdős-Rényi graphs, the spectral radius asymptotically tends to the maximum of ϵ and the square root of the maximum degree of the graph (Krivelevich and Sudakov, 2003). We note that even though range *r* contributes to the value of λ_1_(*G*), its influence is quite small compared to the other parameters. Here, we recall that locality size *s*, λ_1_(*H*_*L*_) and λ_1_(*F*_*LD*_) are factors related to human-mediated dispersal. Under the assumption that the spectral radius is an indicator of rate of dispersal, we can see the significance of these components with respect to the diffusion process. We also note that for larger values of locality size and ϵ, the number of regions *k* contributes to the value of the spectral radius as the maximum degree of *F*_*LD*_ increases with *k*.

While diameter, like spectral radius, is closely related to dynamics, we observe in Figures 3, 4 that the most significant influence on its value is due to the range *r*; this is closely followed by the number of regions *k* and ϵ. We note that doubling the range reduces the diameter by approximately half. Greater the number of regions and ϵ, the greater is the number of long distance edges. Hence, the diameter goes down. We note that unlike the case of spectral radius, locality size is not an important factor with respect to diameter.

### 8.4. Dynamical Analysis of MPSN Model

**Figure 5:**
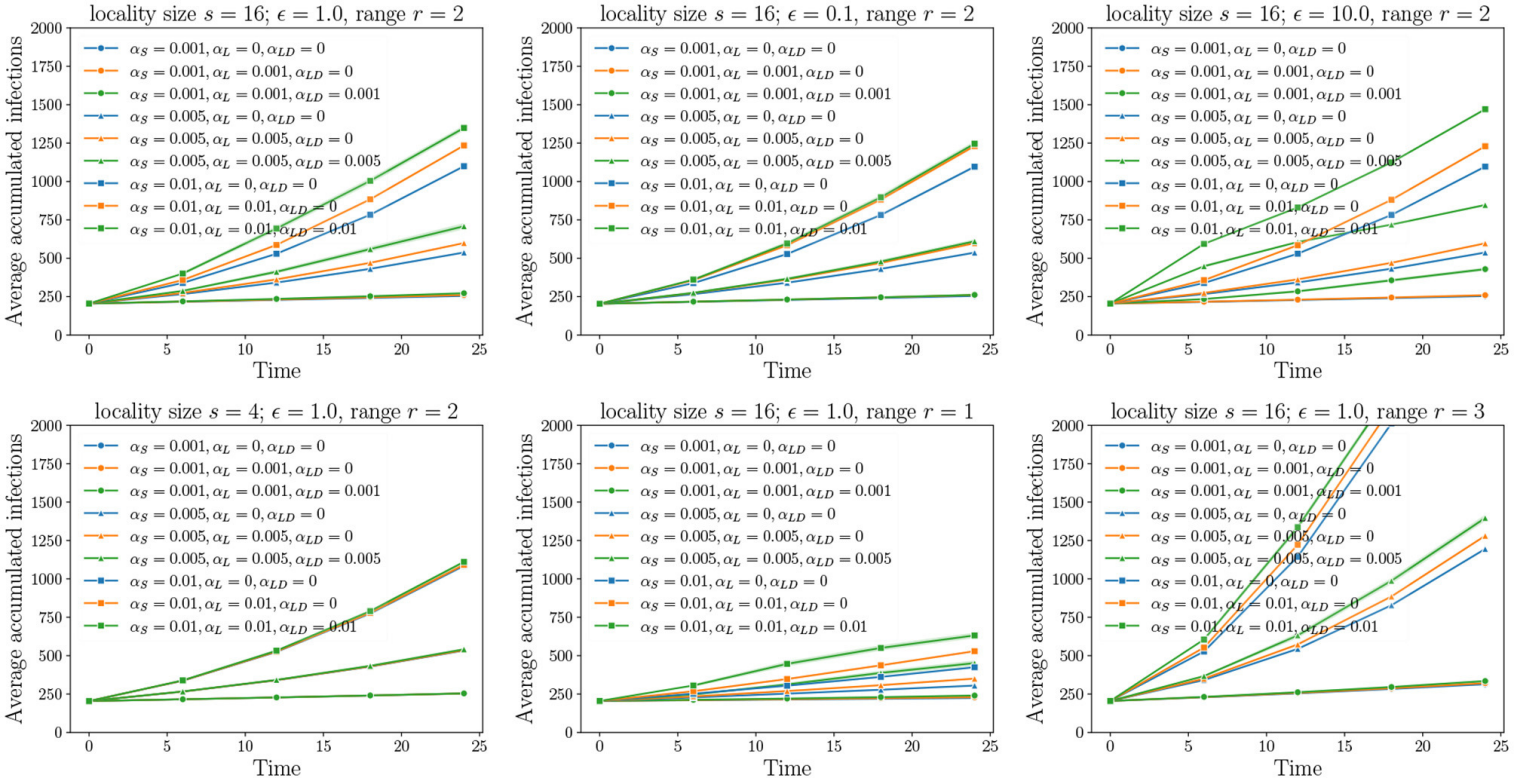
Unraveling pathways: Here, we choose a positive value 0 ≤ *c* < 1. All pathway parameters α*s*, αℓ, and α*ℓd* are either set to 0 or *c*. In the experiments, we incrementally unravel the pathways starting the edges from the grid (α*s* = *c*, αℓ = 0, α*ℓd* = 0), followed by intra-locality edges (α*s* = *c*, αℓ = *c*, α*ℓd* = 0), and finally, inter-locality edges (α*s* = *c*, αℓ = *c*, α*ℓd* = *c*). For all the cases where α*ℓd* ≠ 0, the variance is higher compared to the remaining cases as these also account for 10 replicates of the networks.

**Figure 6:**
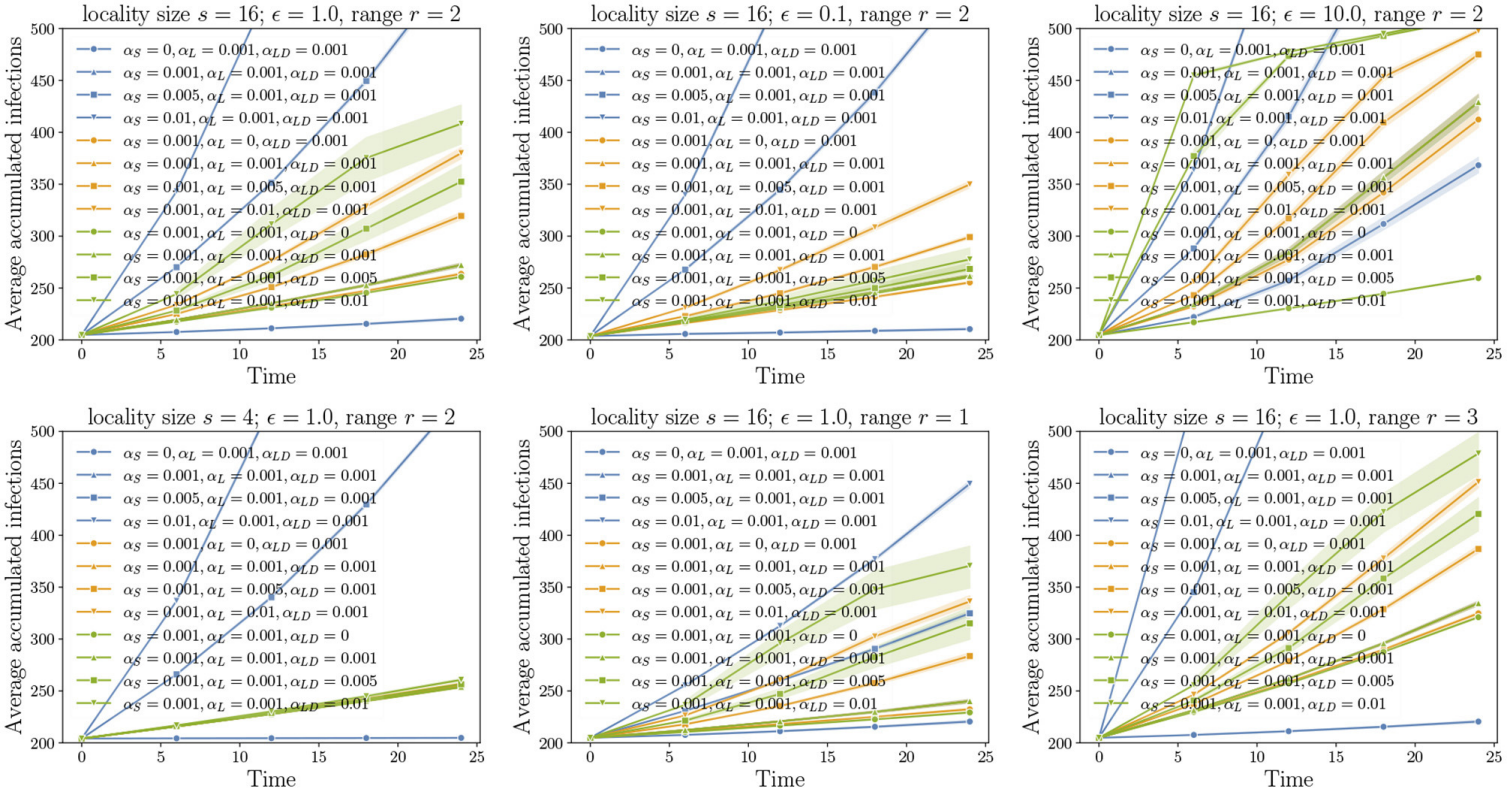
Non-uniform pathway probabilities: In each figure, there are three sets of plots. In each set, two pathway parameters are fixed while the remaining pathway parameter value is varied from 0 to 0.01.

These experiments correspond to diffusion using the SPREAD model on different MPSN instances and different combinations of pathway parameters α*s*, αℓ, and α*ℓd*. The observed variable is the mean number of infections that have occurred by a given time *T*. The results of our experiments are in Figures 5, 6, where the progressions of the diffusion process under different conditions are plotted. A general observation is that, when the number of infected nodes is much greater than $\sqrt{n}$, the rate of spread is linear. This is because, for any infected vertex, most or all of its neighbors are infected. In each time step, at the forefront of the spread process, at most a constant times $\sqrt{n}$ vertices are available to be infected in the next time step.

#### 8.4.1. Progression of the Diffusion Process

Our first set of experiments concern the importance of pathway parameters given a network. Here, we fix a transmission probability on each edge. Using the pathway parameters, we first activate only the edges of *G*_*S*_. This is the baseline process of diffusion over just the grid. From the domain perspective, this corresponds to natural or self-mediated spread alone. Then, we unravel the edges corresponding to *G*_*L*_, followed by the edges of *G*_*LD*_. In Figure 5, the top left plot is the reference plot corresponding to MPSN with *s* = 16, ϵ = 1, and *r* = 2. We see that when only α*s* > 0 or in other words, only the grid edges are activated, the spread is slow in the beginning followed by an increase in the rate before it saturates. A similar phenomenon is observed even when the intra-locality and inter-locality edges are activated. We note that the rate of increase is higher when the inter-locality edges are introduced; this highlights the importance of long distance edges in increasing the rate of spread. We compared the reference plot with diffusion on networks where ϵ = 0.1 (top middle in Figure 5) and ϵ = 10.0 (top right). For ϵ = 0.1, the inter-locality edges do not contribute to the infections, while for ϵ = 10, this pathway is dominant. In addition, the progression of the process is very fast in the beginning and saturates quickly, after which the increase is almost linear. This indicates that the long distance edges contribute to fast short-term spread, an important factor to account for in preparing for interventions.

#### 8.4.2. Importance of Locality Size and Range

The bottom left plot in Figure 5 corresponds to a smaller locality size. We see that locality size significantly affects both intra- and inter-locality spread. Reduction in locality size to 4 makes these pathways insignificant. With increase in range (bottom right), the diffusion through short distance pathway is rapid, and, therefore, the process reaches saturation before either the intra- or inter-locality pathways can make a difference.

#### 8.4.3. Non-Uniform Probabilities

In Figure 6, we fix two pathway parameters while the third parameter is varied. We observe that the highest increase in the rate of spread corresponds to the short-distance parameter α*s* and the least is αℓ, the intra-locality parameter. We also note that larger the ϵ, the greater the number of long distance edges and, therefore, the higher the increase in the rate of spread as α*ℓd* is increased (see top row of Figure 6).

#### 8.4.4. Regression-Tree Analysis

**Figure 7:**
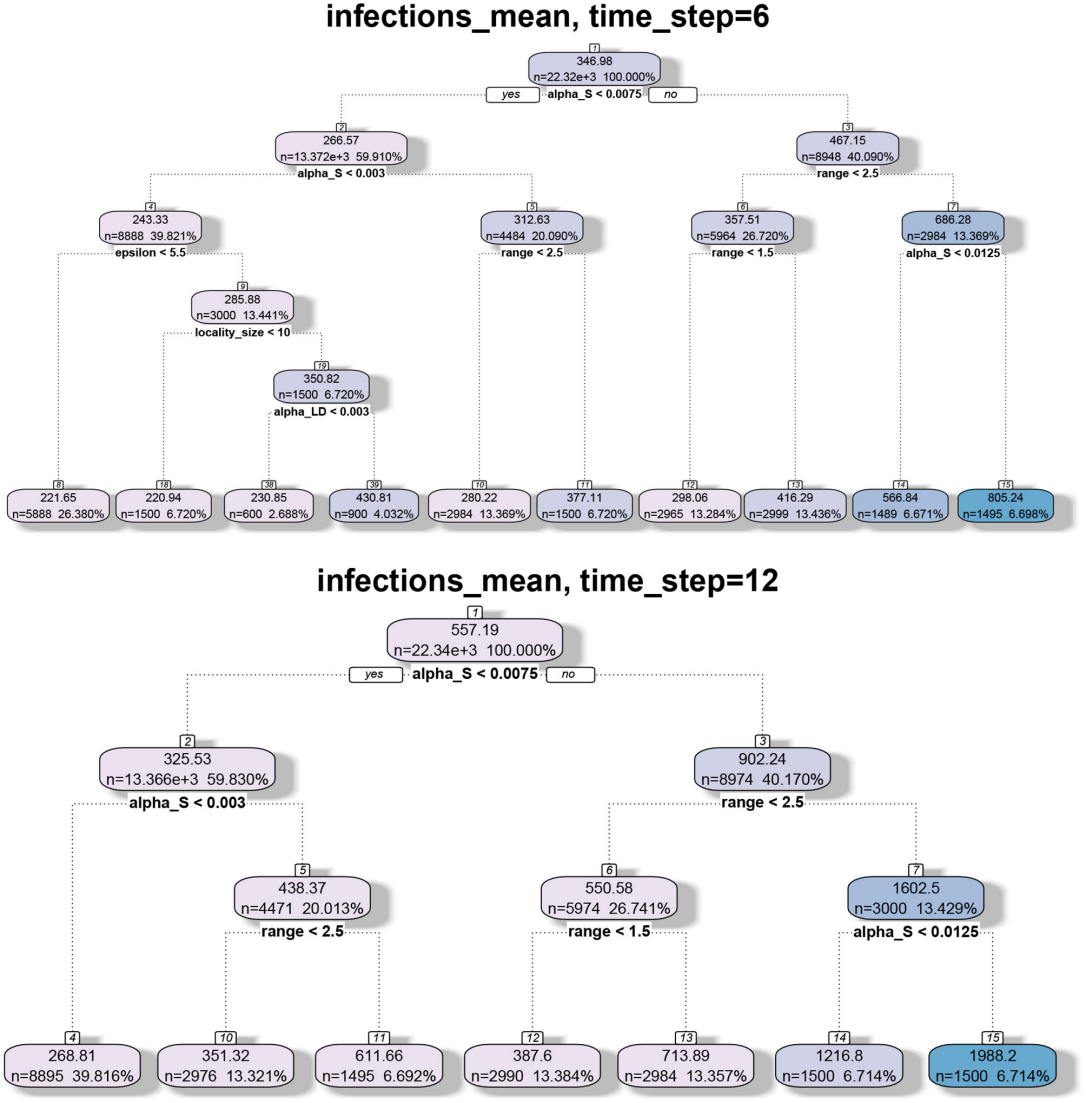
Regression tree analysis of simulation results where the independent variables are the MPSN parameters and pathway parameters of the SPREAD model and the observed variable is the mean number of infections at different time steps *T*.

**Figure 8:**
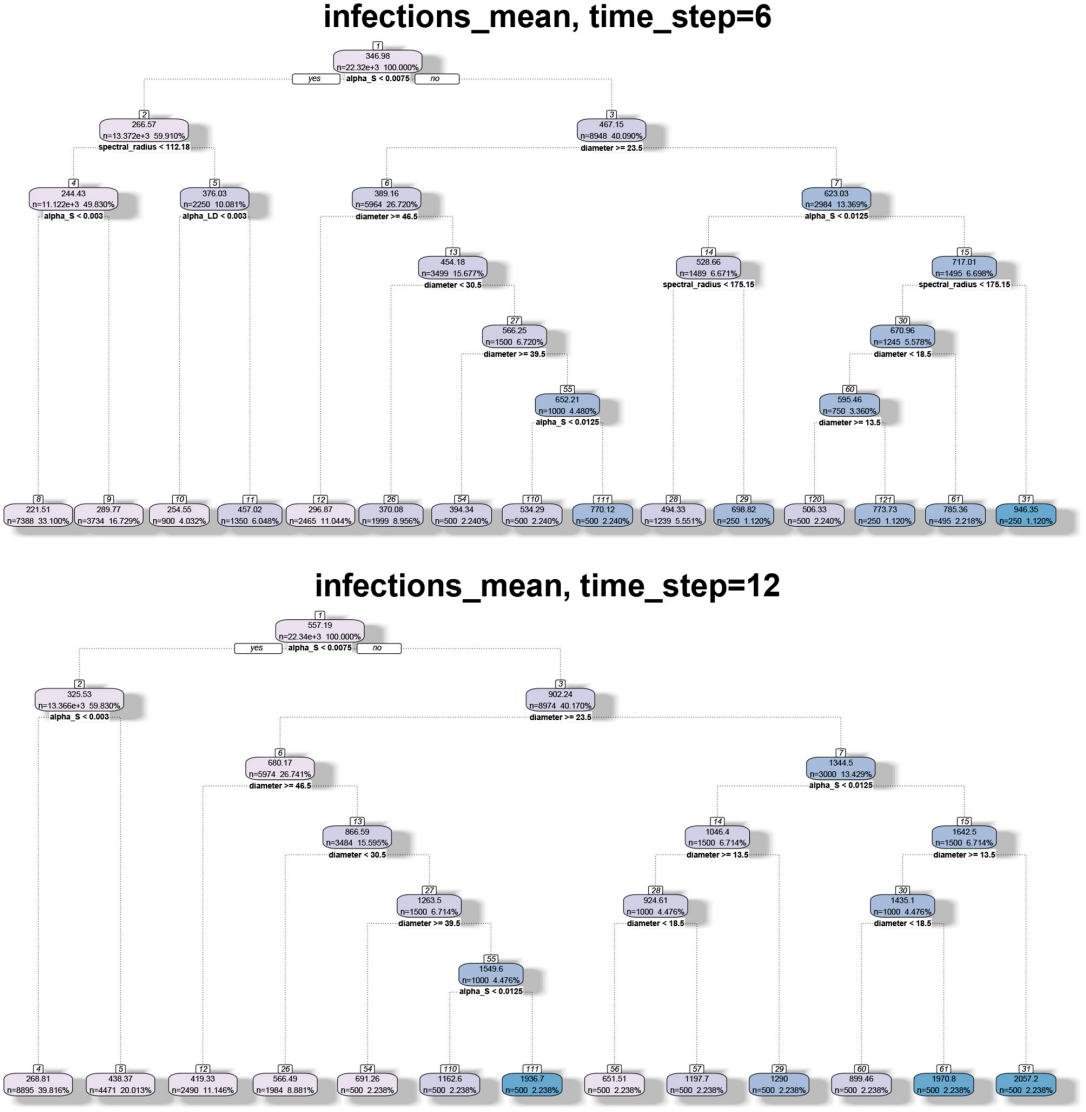
Regression tree analysis of simulation results where the independent variables are the spectral radius and diameter of the networks and the observed variable is the mean number of infections at different time steps *T*.

We applied regression tree and random forest algorithms on the simulation data. The first objective was to find the network and model parameters that drive the infection. The second objective is to determine how structural parameters such as spectral radius and diameter influence the spread. Accordingly, we have two sets of results in Figures 7, 8, respectively. The regression-tree analysis succinctly captures some aspects of the results in Figures 5, 6. As observed in the earlier plots, the rate of spread is determined primarily by α*s* and range *r*. For smaller time steps, the inter-locality spread is significant, particularly when α*s* and *r* are small (Figure 7). Therefore, ϵ and locality size *s* are significant predictors of spread. However, in the later time steps, due to saturation, only the parameters that affect short-distance spread are good predictors of spread.

#### 8.4.5. Spectral Radius, Diameter, and Number of Infections

In Figure 8, we note that spectral radius features only in the first plot. This is again because spectral radius is determined primarily by inter-locality parameters. However, for later time steps, only diameter and α*s* determine the mean number of infections. Again, this just reflects the fact that diameter and range are correlated. Therefore, spectral radius is a good predictor of spread during the initial phase (either first few time steps or when the number of infections is small).

### 8.5. Robustness of Regression Tree Analysis

**Figure 9:**
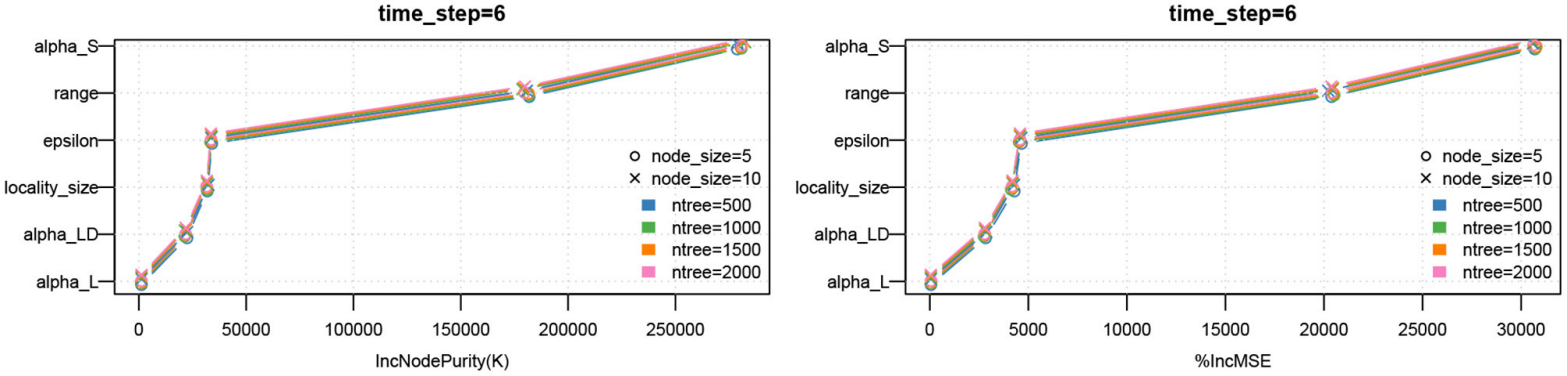
Parameter importance using random forest analysis. The analysis is performed for different hyperparameter values of the random forest model.

We note that the outputs of the random forest algorithm are influenced by hyper parameters, such as number of estimators (or trees), maximum depth, minimum number of samples required to split an internal node, etc. In Figure 9, we have plotted the parameter importance based on increase in node purity (Gini index) and increase in mean square error for node size and number of trees. We note that the results are consistent.

### 8.6. Analysis of Real-World Commodity Flow Networks

For the real-world networks, we computed the spectral radius and diameter for the following graph. We first decomposed the network into 12 components, one for each month of the year. In the SPREAD model of Section 7, we note that in Equations (1)–(3) the probability of transmission is dependent on infectivity ρ(*v, t*). For the short-distance graphs *G*_*S*_ and *G*_*L*_, every outgoing edge was assigned a weight of ρ(*u, t*), where *u* is the source node. For *G*_*LD*_, every outgoing edge was assigned the weight of ρ(*u, t*)*F*_*u**v*_, where *u* is the locality to which the source *u* belongs and *F*_*u**v*_ is the edge weight on the edge {*u*, *v*} in the inter-locality graph *F*_*LD*_. The (weighted) adjacency matrices of these graphs were added together and the spectral radius of the resulting matrix is computed. Note that the spectral radius is a real number as the matrix corresponds to a strongly connected directed graph. This follows from the Perron-Frobenius theorem (Brouwer and Haemers, 2012). We call this the *weighted spectral radius*. We also computed the spectral radius of the graph without the weights (i.e., the weight is 1 iff the *ij*th entry of the adjacency matrix is non-zero). This is referred to as the *unweighted spectral radius*. The diameter was computed for the unweighted graph.

**Figure 10:**
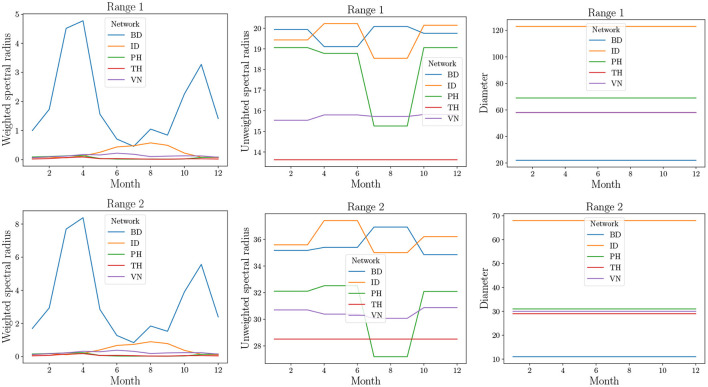
Structural properties of real-world datasets from Table 2. Since these are temporal networks, the properties are plotted for 12 snapshots, each representing a month of the year by natural order. In the top right plot, TH overlaps with VN.

The results are in Figure 10 where the three structural properties are plotted for different months and two range values, namely 1 and 2. We observe that the weighted spectral radius is very high for BD compared to other networks. In all networks, we see variation in the spectral radius. This is due to seasonal fluctuations in the production (which affects ρ) and, therefore, in trade (which affects the long distance edges). The PH network shows highest variation in the case of unweighted spectral radius. This parameter is sensitive to the presence or absence of edges. During offseason, the absence of edges leads to a reduction in the unweighted spectral radius. However, in other networks, during offseason, only the weights decrease. We note that spectral radii increase significantly when the range is increased.

The diameter remains constant and is very high unlike what was observed in MPSN. The primary reason for this is that the trade flows do not follow a random graph pattern. The presence of a few areas of production implies many outgoing edges from a small number of localities to other localities that are major areas of consumption. This implies that the rate of spread could be highly dependent on the seed nodes. Thus, if the seed nodes correspond to high production areas, then the spread will be rapid.

## 9. Summary and Future Work

Motivated by the need to obtain a better understanding of the spread of biological invasions, we proposed an abstract multi-pathway diffusion model. Using structural properties of the underlying networks, we characterized complex spatial diffusion processes over networks. We also investigated the role of the different pathways in determining the rate and pattern of spread. In addition, we analyzed the structural and dynamical properties of networks using data mining algorithms such as regression trees.

Our methods provide just the first step in understanding how multiple pathways and diffusion parameters determine the extent and pattern of the spread of biological invasions. There are several additional directions for future research. First, one can investigate the role of other structural parameters of the graphs generated by multi-pathway model (e.g., clustering coefficient, *k*-core sizes, closeness centrality (Easley and Kleinberg, 2010)) in determining properties of the spread process. A second direction is to investigate the suitability of other epidemic models proposed in the literature (Marathe and Vullikanti, 2013) for biological invasions. A third direction is to incorporate more realistic models like the gravity model and scale-free graphs for inter-locality spread. Finally, it may be of interest to refine the abstract multi-pathway model to allow the generation of temporal networks which play an important role in practice.

## Data Availability Statement

Publicly available datasets were analyzed in this study. This data can be found here at: https://github.com/rmuniappan/SPREAD_model.

## Author Contributions

AA defined the scope of the research. AA, NP, and YB designed and conducted the experiments and analyzed the results. AA, SR, and HM contributed to the theoretical formulation and results and wrote the paper. All authors contributed to the article and approved the submitted version.

## Funding

This work was supported in part by the United States Agency for International Development under the Cooperative Agreement no. AID-OAA-L-15-00001, Feed the Future Innovation Laboratory for Integrated Pest Management, Agricultural AI for Transforming Workforce and Decision Support (AgAID) grant no. 2021-67021-35344 from the USDA National Institute of Food and Agriculture, Network Models of Food Systems and their Application to Invasive Species Spread, grant no. 2019-67021-29933 from the USDA National Institute of Food and Agriculture, UVA Strategic Investment Fund SIF160, NSF Grant IIS-1908530, NSF Expeditions in Computing Grant CCF-1918656, and NSF CINES OAC-1916805.

## Conflict of Interest

The authors declare that the research was conducted in the absence of any commercial or financial relationships that could be construed as a potential conflict of interest.

## Publisher's Note

All claims expressed in this article are solely those of the authors and do not necessarily represent those of their affiliated organizations, or those of the publisher, the editors and the reviewers. Any product that may be evaluated in this article, or claim that may be made by its manufacturer, is not guaranteed or endorsed by the publisher.
